# Supplementary material for: Post-trauma coping in the context of significant adversity: a qualitative study of young people living in an urban township in South Africa
Source: BMJ Open. 2017 Oct 6;7(10):e016560. doi: 10.1136/bmjopen-2017-016560 (PMC5640056; doi:10.1136/bmjopen-2017-016560)
Supplement: Supplementary Table 1 [file bmjopen-2017-016560supp001.pdf]

Supplementary Table 1. Descriptive information.

ID	Gender	Trauma[a]	Total # lifetime traumatic events
A	Female	Violent robbery	5
B	Female	Sexual assault	3
C	Female	Witnessed drowning	3
D	Female	Witnessed brother's beating murder	6
E	Male	Violent house robbery	6
F	Male	Shot by friend (accidental)	4
G	Male	Saw friend shot	3
H	Male	Saw friend shot	2
I	Male	Witnessed beating murder	4
J	Male	Car crashed through house	8
K	Male	Someone shot in home	9
L	Male	Car accident	6
M	Male	Physical assault	8
N	Male	Witnessed friend being stabbed	10
O	Male	Best friend beaten to death	6
P	Male	Witnessed friend's shooting murder	9
Q	Male	Witnessed shooting, then murder	5
R	Female	Raped	4
S	Female	Gang raped	4
T	Female	Raped	3
U	Female	Raped	5
V	Female	Raped	2

W	Female	Raped	8
X	Female	House fire	2
Y	Female	House fire (family member died)	7

[a] Note, ‘Trauma’ identifies the event the young person marked as most upsetting to them *now*. It is not necessarily the most recent traumatic event. Index events had occurred between 2013 and 2016. All had experienced multiple traumatic events and in most interviews the young person would discuss other events, along with the index trauma.
